# Utility of Neck Circumference for Identifying Metabolic Syndrome by Different Definitions in Chinese Subjects over 50 Years Old: A Community-Based Study

**DOI:** 10.1155/2018/3708939

**Published:** 2018-04-12

**Authors:** Shuo Lin, Li Hu, Ping Li, Xiaofeng Li, Keyi Lin, Bilian Zhu, Panwei Mu, Longyi Zeng

**Affiliations:** ^1^Department of Endocrinology, The Third Affiliated Hospital of Sun Yat-sen University, Guangzhou, China; ^2^Department of Endocrinology, The First Hospital of Changsha, Changsha, China; ^3^Department of Gynaecology and Obstetrics, The Third Affiliated Hospital of Sun Yat-sen University, Guangzhou, China; ^4^Department of Pediatrics, The Third Affiliated Hospital of Sun Yat-sen University, Guangzhou, China

## Abstract

**Aims:**

Whether neck circumference (NC) could be used as a valuable tool for identifying metabolic syndrome (MS) by different criteria in Chinese is still unclear.

**Methods:**

We conducted a cross-sectional survey from October 2010 to January 2011 in Shipai community, Guangzhou, Guangdong Province, China. A total of 1473 subjects aged over 50 years were investigated. We measured height, weight, NC, waist circumference, blood pressure, blood glucose, and lipids in all subjects. MS was identified by criteria of the National Cholesterol Education Program-Adult Treatment Panel III (NCEP-ATP III), Chinese Diabetes Society (CDS), and International Diabetes Federation (IDF).

**Results:**

Mean NC was 38.0 ± 2.7 cm in men and 34.2 ± 2.5 cm in women. By using receiver operating characteristic curves, the area under the curve (AUC) of NC for identifying MS (IDF) was 0.823 in men and 0.777 in women, while for identifying MS (CDS), it was 0.788 in men and 0.762 in women. The AUC of NC for diagnosing MS (ATP III) was 0.776 in men and 0.752 in women. The optimal cut points of NC for MS were 38.5 cm by three definitions in men, while those were 34.2 cm, 33.4 cm, and 34.0 cm in women by IDF, ATP III, and CDS definitions, respectively. No significant difference was observed between the AUC of NC and BMI for diagnosing MS by using different criteria (all *p* > 0.05).

**Conclusions:**

NC is associated with MS by different definitions in Chinese subjects over 50 years old. It may be a useful tool to identify MS in a community population.

## 1. Introduction

Metabolic syndrome (MS) was a cluster of cardiovascular risk factors, including obesity, particularly central obesity, diabetes mellitus (DM) or impaired glucose regulation (IGR), dyslipidemia, and raised blood pressure (BP) [1]. There were many diagnostic criteria of MS. Of them, the definitions of the National Cholesterol Education Program-Adult Treatment Panel III (NCEP-ATP III) [2] and International Diabetes Federation (IDF) [3] were widely used. In China, we used the MS definition of the Chinese Diabetes Society (CDS) [4] (revised in 2013) as the diagnostic criterion. Although there were still various definitions of MS, the associations of MS with cardiovascular diseases and diabetes have been well established [1–3]. Recent studies showed that different fat distribution may be associated with different metabolic risks [5]. Body mass index (BMI) is a useful index of whole-body adiposity. Waist circumference (WC) is widely used to define central obesity for its correspondence to abdominal visceral fat [6, 7] and is considered as the key component of MS in several diagnostic criteria [1–3].

Despite the common usage of WC for identification of obesity and MS, it has some limitations. First, different studies reported various ways to determine the specific site for measuring WC, which may influence the measured WC values [8]. Second, measuring WC may not be accurate and convenient for large population investigations, especially in cold weather and heavy clothing. Last but not least, it has interday variations and may be influenced by changes of the abdominal wall and abdominal cavity.

Neck circumference (NC), a new anthropometric parameter representative of upper-body adiposity, has been reported to be more convenient for screening overweight or obesity than WC [9–14]. Moreover, recent studies also suggested NC was positively related with central obesity and metabolic syndrome [9–12]. However, whether it can be applied in Chinese over 50 years old by using different MS definitions, as well as the optimal cut points to diagnose MS, needs further investigation. This study is aimed at exploring the relationship between NC and MS and its components in Chinese subjects over 50 years old in a community setting.

## 2. Subjects and Methods

We conducted a community-based cross-sectional survey from October 2010 to January 2011 in Shipai community, Guangzhou, Guangdong Province, China. Details of the study have been published elsewhere [15, 16]. All subjects over 50 years old (*n* = 2052) in this community were invited to participate in the survey. Subjects with medical illnesses such as clinical or laboratory evidence of cardiac, renal, liver, or endocrine disease, severe systemic diseases, and patients with thyromegaly were excluded. The survey was approved by the ethics committee of the Third Affiliated Hospital of Sun Yat-sen University. Written informed consent was obtained from all participants before the survey.

### 2.1. Assessment

All subjects completed a questionnaire containing information of the medical history and a physical examination including measuring of height, weight, WC, NC, and blood pressure as described before [15, 16]. Fasting venous samples were obtained to measure blood lipids, plasma glucose, insulin, and uric acid.

NC was measured with the head erect and eyes facing forward, horizontally at the upper margin of the thyroid cartilage (to the nearest 0.1 cm) by a trained physician as other studies described [9–11, 17]. Detailed anthropometry (height, weight, and waist circumference) and blood pressure were measured by methods as previously described [15, 16]. We measured plasma glucose by the glucose oxidase method. We measured blood lipids and uric acid by using HITACHI 7180 (Hitachi High-Tech Science Systems Corporation, Hitachinaka-shi, Japan).

Hypertension was defined as systolic blood pressure (SBP) ≥ 140 mmHg and/or diastolic blood pressure (DBP) ≥90 mmHg or hypertension history diagnosed by a physician or by being on antihypertensive medication. MS was diagnosed based on three definitions, the NCEP-ATP III [2] (central obesity was defined as WC ≥ 90.0 cm for men and WC ≥ 80.0 cm for women in Chinese), Chinese Diabetes Society (CDS) [4], and International Diabetes Federation (IDF) [3] (central obesity was defined as WC ≥ 90.0 cm for men and WC ≥ 80.0 cm for women in Chinese) (Supplementary Table 1). In detail, the definition of MS (CDS) requires the presence of any three or more of the following five compositions [4]: (1) central obesity, defined as WC ≥ 90.0 cm for men and WC ≥ 85.0 cm for women in Chinese; (2) hyperglycemia, defined as a FPG ≥ 6.1 mmol/L, a 2hPG ≥ 7.8 mmol/L, and (or) previously diagnosed diabetes; (3) hypertension, defined as SBP ≥ 130 mmHg, DBP ≥ 85 mmHg, and (or) previously diagnosed hypertension; (4) hypertriglyceridemia, defined as a serum triglyceride (TG) ≥ 1.70 mmol/L; (5) low high-density lipoprotein cholesterol (HDL-C), defined as a serum HDL-C less than 1.04 mmol/L.

### 2.2. Statistical Analysis

We used SPSS for Windows 19.0 for statistical analysis. Continuous variables are presented as means (SD) or medians (interquartile range) for skewed variables. We compared differences in continuous variables between groups by independent *t*-test (assuming a Gaussian distribution) or Mann–Whitney *U* test (assuming a non-Gaussian distribution). We used the Pearson correlation to explore the association of NC with other factors. The receiver operating characteristic (ROC) curve of BMI and NC was plotted to determine the optimal threshold for predicting MS. We considered a *p* value < 0.05 as statistically significant for a two-sided test.

## 3. Results

### 3.1. Clinical Characteristics of the Study Participants

We recruited 1494 subjects (72.8%) in this survey. After excluding 21 participants with missing data, 1473 subjects had a median age of 61 years (interquartile range: 55–68 years) and mean FPG 5.50 ± 2.00 mmol/L and were 61.4% female (Table 1). The mean NC value was 38.0 ± 2.7 cm in men and 34.2 ± 2.5 cm in women. Men were heavier; had higher levels of WC, NC, and uric acid; and had lower levels of BMI, SBP, total cholesterol, LDL cholesterol, HDL cholesterol, and LnHOMA-IR. The distribution of age and sex in subjects who did not participate in the survey was similar to that of the participants, which indicated that the investigated population was representative.

### 3.2. Correlations of NC with Anthropometric and Laboratory Parameters

Table 2 shows the correlation of neck NC with anthropological and metabolic variables in men and women. NC was significantly positively correlated with WC (correlation coefficient was 0.775 in men and 0.732 in women, *p* < 0.001) and body mass index (correlation coefficient was 0.767 in men and 0.735 in women, *p* < 0.001). In addition, NC was positively correlated with SBP, DBP, FBG, TG, uric acid, and LnHOMA-IR in both men and women. In contrast, NC was negatively correlated with HDL-C in both genders. No significant correlation was found between NC and TC and LDL-C in women.

### 3.3. Optimal Cutoff Points of NC and BMI for Identifying MS by Different Criteria

**Figure 1:**
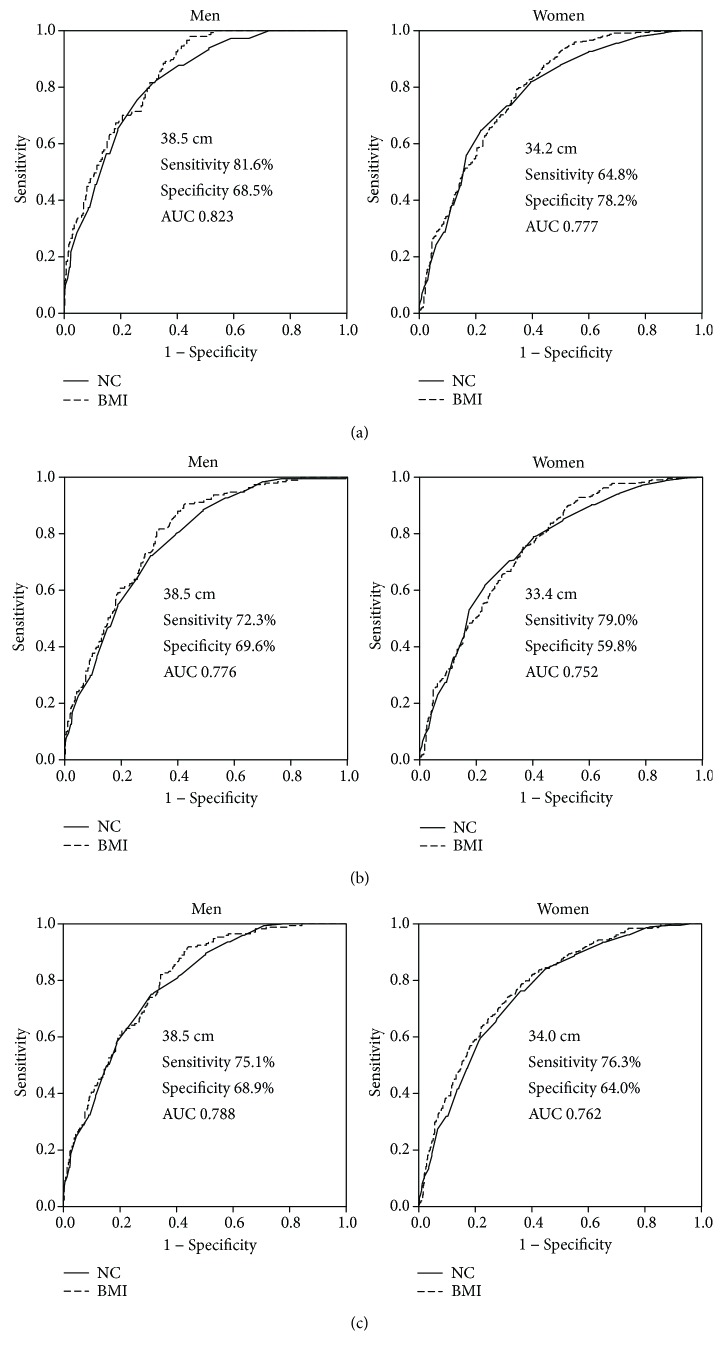
Receiver operating characteristic curves of neck circumference and body mass index for identifying metabolic syndrome in men and women. (a) MS (IDF); (b) MS (ATP III); (c) MS (CDS).

ROC curves were plotted to analyze the optimal NC cutoffs for identifying MS with IDF, NCEP-ATP III, and CDS definitions (Figure 1). The area under the curves (AUC) of NC for diagnosing MS were 0.823 (95% CI: 0.789~0.853) for men and 0.777 (95% CI: 0.748~0.803) for women with the IDF definition. NC ≥ 38.5 cm for men and ≥34.2 cm for women were the best cutoff points for determining subjects with MS. The AUC of BMI for diagnosing MS were 0.846 (0.814~0.875) in men and 0.790 (0.762~0.816) in women. With the NCEP-ATP III definition, the AUC of NC for MS was 0.776 (95% CI: 0.739~0.809) for men and 0.752 (95% CI: 0.722~0.780) for women. The optimal cutoff points of NC were 38.5 cm in men and 33.4 cm in women. The AUC of BMI for diagnosing MS were 0.797 (0.762~0.829) in men and 0.756 (0.726~0.783) in women. According to the CDS definition, the AUC were 0.788 (95% CI: 0.752~0.821) and 0.762 (95% CI: 0.733~0.789) in men and women, respectively. The optimal cutoff points were 38.5 cm in men and 34.0 cm in women (Table 3). The AUC of BMI for diagnosing MS were 0.801 (0.766~0.833) in men and 0.780 (0.752~0.807) in women. No significant difference was observed between the AUC of NC and BMI for diagnosing MS by using different criteria (all *p* > 0.05).

In males, the AUC of WC for diagnosing MS (IDF), MS (ATP III), and MS (CDS) were 0.923, 0.838, and 0.849, respectively (Table 3). In females, the AUC of WC for diagnosing MS (IDF), MS (ATP III), and MS (CDS) were 0.839, 0.799, and 0.833, respectively. The AUC of WC for diagnosing MS were larger than those of NC or BMI (all *p* < 0.05, Table 3), by using three different criteria.  Table 4 shows the comparison of NC and WC for discriminating MS (ATP III) and its components. In males, NC ≥ 38.5 cm showed a higher sensitivity and a lower specificity in detecting hypertension, hypertriglyceridemia, low HDL-C, and hyperglycemia, compared with WC. Similar sensitivity and specificity were observed for diagnosing MS (ATP III) by NC or WC. In females, NC ≥ 33.4 cm showed a lower sensitivity and a higher specificity in detecting hypertension, hypertriglyceridemia, low HDL-C, hyperglycemia, and MS (ATP III), compared with WC.

## 4. Discussion

In the cross-sectional survey, we demonstrated that neck circumference was a good anthropometric marker of MS by using different criteria in Chinese subjects. According to ROC curves, a NC ≥ 38.5 cm was the optimal threshold for diagnosing MS in men by using the definitions of ATP III, CDS, and IDF, while a NC of 33.4–34.2 cm showed the best diagnostic accuracy for diagnosing MS by the three definitions in women. The diagnostic accuracy of NC for detecting MS was similar to BMI irrespective of various criteria. Our results suggested NC may be a useful tool to identify MS in Chinese subjects over 50 years in a community setting.

Several studies have reported the use of NC for identifying obesity and MS by various criteria in different populations [9–11, 13, 18–21]. Yang et al. reported that NC was significantly positively related with BMI and WC in Chinese subjects with type 2 diabetes and a NC of ≥39 cm for men and ≥35 cm for women was the best cutoff point for diagnosing MS (CDS 2004) [10]. A study conducted in elder Chinese reported that the optimal cutoff points of neck circumference for MS (by CDS 2004) were 38 cm in men (the AUC was 0.76) and 35 cm in women (the AUC was 0.73) [11]. Bao et al. reported that the AUC of NC to determine visceral adiposity (quantified by magnetic resonance imaging) was 0.781 for men and 0.777 for women in a community population. The optimal cutoffs for identifying visceral obesity were 38.5 cm in men and 34.5 cm in women. There were no significant differences between the proportions of MS and its components identified by an increased NC and WC [9]. In accordance with these studies, our results suggested that NC may be used for identifying obesity and MS in Chinese. The optimal cut points were very close to those of these studies. Different studies reported different NC cut points possibly due to different study populations and diagnostic criteria. Further prospective studies need to be conducted to explore the optimal cut points for specific population.

In this study, by using the IDF, NCEP-ATP III, or CDS criteria, men had the same optimal cut points of 38.5 cm for diagnosing MS, while the optimal cut points were 33.4–34.2 cm in women (IDF > CDS > ATP III). The AUC of NC for diagnosing MS by different criteria varied from 0.776 to 0.823 in men and 0.752 to 0.777 in women. No significant difference was observed between the AUC of NC and BMI for diagnosing MS by different criteria (all *p* > 0.05). However, the AUC of WC for diagnosing MS were larger than those of NC or BMI, by using three different criteria, suggesting that WC yielded higher diagnostic accuracy for identifying MS than NC or BMI. A possible explanation might be that WC is one of the criteria of MS. These results showed NC and BMI had similar diagnostic accuracy for diagnosing MS. Given that NC is simple, invariable, repeatable, and inexpensive, and sometimes it might be a better index for adverse risk profile than WC [9, 20, 22, 23], NC could be used as an alternative tool for diagnosing MS in Chinese subjects over 50 years old, irrespective of various diagnostic criteria of MS.

Our study adds to the literature by showing that NC could be used as a valuable tool for identifying MS in a large community-based cohort. In addition, to our knowledge, we first compared the diagnostic accuracy of NC and BMI for identifying MS by different criteria in a community-based population. There were some limitations in our study. First, our study only investigated community subjects over 50 years old in south China, which may restrict the application of our conclusion. Further studies need to be conducted in other populations in China. Second, our study is a cross-sectional study, so we could not explore the ability of NC for predicting cardiovascular events or other clinical outcomes. We need to evaluate the accuracy of NC for predicting cardiovascular events by prospective cohort studies.

In conclusion, as well as BMI, NC is associated with MS by using different definitions in Chinese community subjects over 50 years old. It may be a useful screening tool to identify adults over 50 years old with MS.

## Figures and Tables

**Table 1:** Clinical characteristics of study subjects.

	Total	Men (*n* = 569)	Women (*n* = 904)	*p*
Age	61.0 (55.0–68.0)	59.0 (54.0–65.0)	62.0 (55.0–69.0)	<0.001
WC (cm)	86.7 ± 9.2	87.43 ± 9.02	86.21 ± 9.28	<0.001
BMI (kg/m^2^)	24.30 ± 3.50	24.05 ± 3.25	24.43 ± 3.63	0.038
NC (cm)	35.7 ± 3.2	38.2 ± 2.7	34.2 ± 2.5	<0.001
SBP (mmHg)	137 ± 21	132 ± 19	139 ± 21	<0.001
DBP (mmHg)	81 ± 10	81 ± 10	81 ± 10	0.313
FBG (mmol/L)	5.50 ± 2.00	5.48 ± 2.02	5.46 ± 2.00	0.297
TC (mmol/L)	5.87 ± 1.16	5.63 ± 1.11	6.03 ± 1.16	<0.001
HDL-c (mmol/L)	1.45 ± 0.36	1.35 ± 0.34	1.51 ± 0.36	<0.001
TG (mmol/L)	1.88 (1.33–2.80)	1.86 (1.32–2.96)	1.90 (1.33–2.76)	0.738
LDL-c (mmol/L)	3.64 ± 0.98	3.49 ± 0.94	3.74 ± 1.00	<0.001
Uric acid (μmol/L)	373.4 ± 108.1	406.2 ± 105.5	352.8 ± 104.6	<0.001
LnHOMA-IR	0.64 ± 0.72	0.43 ± 0.70	0.78 ± 0.69	<0.001

WC: waist circumference; BMI: body mass index; NC: neck circumference; SBP: systolic blood pressure; DBP: diastolic blood pressure; FBG: fasting blood glucose; TC: total cholesterol; HDL-c: high-density lipoprotein cholesterol; TG: triglyceride; LDL-c: low-density lipoprotein cholesterol; LnHOMA-IR: natural logarithm of homeostasis model assessment of insulin resistance.

**Table 2:** Correlation of neck circumference with anthropological and metabolic variables in men and women.

Variable	Men (*n* = 569)		Women (*n* = 904)	
	*r*	*p*	*r*	*p*
WC (cm)	0.775	<0.001	0.732	<0.001
BMI (kg/m^2^)	0.767	<0.001	0.735	<0.001
SBP (mmHg)	0.257	<0.001	0.209	<0.001
DBP (mmHg)	0.275	<0.001	0.214	<0.001
FBG (mmol/L)	0.109	<0.001	0.213	<0.001
TG (mmol/L)	0.378	<0.001	0.274	<0.001
TC (mmol/L)	0.210	<0.001	0.026	0.438
LDL-C (mmol/L)	0.122	0.004	0.015	0.649
HDL-C (mmol/L)	−0.287	<0.001	−0.279	<0.001
Uric acid (μmol/L)	0.133	0.002	0.301	<0.001
LnHOMA-IR	0.459	<0.001	0.536	<0.001

WC: waist circumference; BMI: body mass index; SBP: systolic blood pressure; DBP: diastolic blood pressure; FBG: fasting blood glucose; TG: triglyceride; TC: total cholesterol; LDL-c: low-density lipoprotein cholesterol; HDL-c: high-density lipoprotein cholesterol; LnHOMA-IR: natural logarithm of homeostasis model assessment of insulin resistance.

**Table 3:** Optimal cut points and the AUC of NC, BMI, or WC for MS according to three definitions.

Criterion		Men				Women			
		Cut point	AUC	95% CI	*p*	Cut point	AUC	95% CI	*p*
IDF	NC	38.5 cm	0.823 ^∗∗^	0.789~0.853	<0.0001	34.2 cm	0.777^∗∗^	0.748~0.803	<0.0001
	BMI	23.5 kg/m^2^	0.846 ^∗∗^	0.814~0.875	<0.0001	23.5 kg/m^2^	0.790^∗∗^	0.762~0.816	<0.0001
	WC	90.0 cm	0.923	0.898~0.944	<0.0001	85.5 cm	0.839	0.813~0.862	<0.0001
ATP III	NC	38.5 cm	0.776^∗∗^	0.739~0.809	<0.0001	33.4 cm	0.752^∗^	0.722~0.780	<0.0001
	BMI	24.1 kg/m^2^	0.797^∗^	0.762~0.829	<0.0001	23.5 kg/m^2^	0.756^∗∗^	0.726~0.783	<0.0001
	WC	89.5 cm	0.838	0.806~0.868	<0.0001	85.7 cm	0.799	0.771~0.824	<0.0001
CDS	NC	38.5 cm	0.788^∗^	0.752~0.821	<0.0001	34.0 cm	0.762^∗∗^	0.733~0.789	<0.0001
	BMI	24.1 kg/m^2^	0.801^∗^	0.766~0.833	<0.0001	24.3 kg/m^2^	0.780^∗∗^	0.752~0.807	<0.0001
	WC	90.0 cm	0.849	0.817~0.877	<0.0001	85.7 cm	0.833	0.807~0.857	<0.0001

^∗∗^ *p* < 0.0001 compared with the AUC of WC by the same definition; ^∗^ *p* < 0.01 compared with the AUC of WC by the same definition. NC: neck circumference; BMI: body mass index; AUC: area under the curve; CI: confidence interval; IDF: International Diabetes Federation; ATP III: Adult Treatment Panel III; CDS: Chinese Diabetes Society.

**Table 4:** Comparison of NC and WC for discriminating metabolic syndrome and its components.

	Sensitivity (%)		Specificity (%)		PPV (%)		NPV (%)	
Men	NC ≥ 38.5 cm	WC ≥ 90 cm	NC ≥ 38.5 cm	WC ≥ 90 cm	NC ≥ 38.5 cm	WC ≥ 90 cm	NC ≥ 38.5 cm	WC ≥ 90 cm
Hypertension	50.7 (45.3–56.2)	45.4 (40.4–50.9)	64.7 (58.1–70.8)	68.5 (62.1–74.5)	67.6 (61.4–73.3)	67.7 (61.2–73.7)	47.5 (41.9–53.1)	46.4 (41.0–51.8)
Hypertriglyceridemia	56.1 (50.5–61.6)	48.9 (43.3–54.4)	70.6 (64.5–76.2)	72.2 (66.2–77.7)	71.1 (65.1–76.7)	69.5 (63.0–75.4)	55.4 (49.7–60.9)	52.2 (46.8–57.6)
Low HDL-C	69.3 (58.6–78.7)	63.6 (52.7–73.6)	60.1 (55.6–84.5)	64.7 (60.2–68.9)	24.1 (19.0–29.9)	24.8 (19.3–30.9)	91.5 (87.8–94.3)	90.7 (87.1–93.5)
Hyperglycemia	56.1 (48.3–63.6)	52.0 (44.3–59.7)	60.6 (55.6–65.4)	65.7 (60.7–70.3)	38.3 (32.3–44.7)	39.8 (33.4–46.5)	75.9 (70.8–80.6)	75.8 (70.9–80.2)
MS (ATP III)	72.3 (65.3–78.5)	76.4 (69.8–82.3)	69.6 (64.7–74.2)	78.8 (74.4–82.8)	54.5 (48.2–60.8)	64.6 (58.0–70.8)	83.2 (78.6–87.2)	86.9 (82.8–90.3)
Women	NC ≥ 33.4 cm	WC ≥ 80 cm	NC ≥ 33.4 cm	WC ≥ 80 cm	NC ≥ 33.4 cm	WC ≥ 80 cm	NC ≥ 33.4 cm	WC ≥ 80 cm
Hypertension	68.1 (64.3–71.8)	80.7 (77.4–83.8)	51.8 (45.8–57.7)	36.0 (30.4–41.9)	75.3 (71.5–78.8)	73.2 (69.7–76.5)	42.9 (37.6–48.3)	46.4 (39.7–53.2)
Hypertriglyceridemia	70.7 (66.6–74.6)	83.3 (79.9–86.4)	50.3 (45.1–55.4)	35.3 (30.5–40.4)	66.0 (61.9–69.9)	63.8 (60.0–67.4)	55.7 (50.2–61.0)	60.8 (54.0–67.3)
Low HDL-C	75.2 (69.5–80.3)	86.6 (81.9–90.5)	43.6 (39.7–47.5)	29.1 (25.6–32.8)	35.2 (31.3–39.4)	33.3 (29.8–37.0)	81.2 (76.6–85.1)	84.2 (78.7–88.8)
Hyperglycemia	77.1 (72.2–81.6)	87.5 (83.4–90.9)	46.9 (42.7–51.0)	31.4 (27.6–35.4)	45.3 (41.1–49.5)	42.1 (38.3–45.9)	78.3 (73.5–82.5)	81.5 (75.8–86.4)
MS (ATP III)	79.0 (75.1–82.4)	94.4 (92.1–96.3)	59.8 (54.8–64.6)	48.5 (43.5–53.5)	71.2 (67.2–74.9)	69.8 (66.2–73.2)	69.3 (64.1–74.1)	87.4 (82.3–91.5)

NC: neck circumference; WC: waist circumference; PPV: positive predictive value; NPV: negative predictive value; HDL-C: high-density lipoprotein cholesterol; ATP III: Adult Treatment Panel III.

## Supplementary Materials

Supplementary Materials Table 1: comparison of three definitions of metabolic syndrome.
