# Supplementary material for: Utility of Neck Circumference for Identifying Metabolic Syndrome by Different Definitions in Chinese Subjects over 50 Years Old: A Community-Based Study
Source: J Diabetes Res. 2018 Apr 12;2018:3708939. doi: 10.1155/2018/3708939 (PMC5925132; doi:10.1155/2018/3708939)
Supplement: Supplementary Materials — Table 1: comparison of three definitions of metabolic syndrome. [file 3708939.f1.docx]

|  | NCEP-ATP III (2005) | CDS（2013） | IDF（2005） |
| --- | --- | --- | --- |
| Number of metabolic components | ≥3 | ≥3 | ≥3 (including central obesity) |
| Central obesity | not necessary | not necessary | necessary |
| WC (cm) | Population specific cut-point  Male≥90cm,Female≥80cm(Chinese) | Male≥90cm,Female≥85cm | Population specific cut-point  Male≥90cm,Female≥80cm(Chinese) |
| Dyslipidemia |  |  |  |
| TG (mmol/L)  HDL-c (mmol/L) | ≥1.7  ＜1.04 (male) or  ＜1.30 (female) | ≥1.7  ＜1.04 (male or female) | ≥1.7  ＜1.04 (male) or  ＜1.30 (female) |
| Elevated blood pressure  (mmHg) | SBP/DBP≥130/85  or hypertension | SBP/DBP≥130/85  or hypertension | SBP/DBP≥130/85  or hypertension |
| Hyperglycemia | FPG≥5.6mmol/L  or diagnosed diabetes | FPG≥6.1mmol/L  or 2h-PBG≥7.8mmol/L or diagnosed diabetes | FPG≥5.6mmol/L  or diagnosed diabetes |

Supplementary table 1. Comparison of three definition of metabolic syndrome

NCEP-ATPIII National cholesterol education program - Adult Treatment Panel III, CDS Chinese Diabetes Society, IDF International Diabetes Federation, WC waist circumference, TG Triglyceride, HDL-c high density lipoprotein cholesterol, SBP systolic blood pressure，DBP diastolic blood pressure，FPG fasting plasma glucose
